# Radioimmunotherapy of PANC-1 human pancreatic cancer xenografts in NOD/SCID or NRG mice with Panitumumab labeled with Auger electron emitting, ^111^In or β-particle emitting, ^177^Lu

**DOI:** 10.1186/s41181-020-00111-y

**Published:** 2020-11-09

**Authors:** Sadaf Aghevlian, Zhongli Cai, David Hedley, Mitchell A. Winnik, Raymond M. Reilly

**Affiliations:** 1 Department of Pharmaceutical Sciences, Leslie Dan Faculty of Pharmacy, University of Toronto, 144 College Street, Toronto, Ontario M5S 3M2 Canada; 2 Department of Chemistry, University of Toronto, 80 St. George St, Toronto, Ontario M5S 3H6 Canada; 3 Department of Medical Oncology, Princess Margaret Cancer Centre, University Health Network, Toronto, ON Canada; 4 Department of Medical Imaging, University of Toronto, Toronto, ON Canada

**Keywords:** EGFR, Panitumumab, Pancreatic cancer, ^111^In, ^177^Lu

## Abstract

**Background:**

Epidermal growth factor receptors (EGFR) are overexpressed on > 90% of pancreatic cancers (PnCa) and represent an attractive target for the development of novel therapies, including radioimmunotherapy (RIT). Our aim was to study RIT of subcutaneous (s.c.) PANC-1 human PnCa xenografts in mice using the anti-EGFR monoclonal antibody, panitumumab labeled with Auger electron (AE)-emitting, ^111^In or β-particle emitting, ^177^Lu at amounts that were non-toxic to normal tissues.

**Results:**

Panitumumab was conjugated to DOTA chelators for complexing ^111^In or ^177^Lu (panitumumab-DOTA-[^111^In]In and panitumumab-DOTA-[^177^Lu]Lu) or to a metal-chelating polymer (MCP) with multiple DOTA to bind ^111^In (panitumumab-MCP-[^111^In]In). Panitumumab-DOTA-[^177^Lu]Lu was more effective per MBq exposure at reducing the clonogenic survival in vitro of PANC-1 cells than panitumumab-DOTA-[^111^In]In or panitumumab-MCP-[^111^In]In. Panitumumab-DOTA-[^177^Lu]Lu caused the greatest density of DNA double-strand breaks (DSBs) in the nucleus measured by immunofluorescence for γ-H2AX. The absorbed dose in the nucleus was 3.9-fold higher for panitumumab-DOTA-[^177^Lu]Lu than panitumumab-DOTA-[^111^In]In and 7.7-fold greater than panitumumab-MCP-[^111^In]In. No normal tissue toxicity was observed in NOD/SCID mice injected intravenously (i.v.) with 10.0 MBq (10 μg; ~ 0.07 nmoles) of panitumumab-DOTA-[^111^In]In or panitumumab-MCP-[^111^In]In or in NRG mice injected i.v. with 6.0 MBq (10 μg; ~ 0.07 nmoles) of panitumumab-DOTA-[^177^Lu]Lu. There was no decrease in complete blood cell counts (CBC) or increased serum alanine aminotransferase (ALT) or creatinine (Cr) or decreased body weight. RIT inhibited the growth of PANC-1 tumours but a 5-fold greater total amount of panitumumab-DOTA-[^111^In]In or panitumumab-MCP-[^111^In]In (30 MBq; 30 μg; ~ 0.21 nmoles) administered in three fractionated amounts every three weeks was required to achieve greater or equivalent tumour growth inhibition, respectively, compared to a single amount of panitumumab-DOTA-[^177^Lu]Lu (6 MBq; 10 μg; ~ 0.07 nmoles). The tumour doubling time (TDT) for NOD/SCID mice with s.c. PANC-1 tumours treated with panitumumab-DOTA-[^111^In]In or panitumumab-MCP-[^111^In]In was 51.8 days and 28.1 days, respectively. Panitumumab was ineffective yielding a TDT of 15.3 days vs. 15.6 days for normal saline treated mice. RIT of NRG mice with s.c. PANC-1 tumours with 6.0 MBq (10 μg; ~ 0.07 nmoles) of panitumumab-DOTA-[^177^Lu]Lu increased the TDT to 20.9 days vs. 11.5 days for panitumumab and 9.1 days for normal saline. The absorbed doses in PANC-1 tumours were 8.8 ± 3.0 Gy and 2.6 ± 0.3 Gy for panitumumab-DOTA-[^111^In]In and panitumumab-MCP-[^111^In]In, respectively, and 11.6 ± 4.9 Gy for panitumumab-DOTA-[^177^Lu]Lu.

**Conclusion:**

RIT with panitumumab labeled with Auger electron-emitting, ^111^In or β-particle-emitting, ^177^Lu inhibited the growth of s.c. PANC-1 tumours in NOD/SCID or NRG mice, at administered amounts that caused no normal tissue toxicity. We conclude that EGFR-targeted RIT is a promising approach to treatment of PnCa.

**Supplementary Information:**

**Supplementary information** accompanies this paper at 10.1186/s41181-020-00111-y.

## Background

Pancreatic cancer (PnCa) is one of the most lethal cancers since most patients are diagnosed when there is local invasion or metastasis, rendering these patients ineligible for surgical treatment (Kamisawa et al., 2016). Surgery provides the only opportunity for long-term survival in PnCa (Kamisawa et al., 2016). The median survival for patients receiving surgery is 17–23 months while those with locally advanced PnCa or metastases have a median survival of 8–14 and 4–6 months, respectively (Cleary et al., 2004). Modest improvements in patient outcome have been achieved with the introduction of FOLFIRINOX, a potent chemotherapy regimen that combines oxaliplatin, irinotecan, leucovorin and 5-fluorouracil but this treatment is associated with high toxicity (Conroy et al., 2011). Thus, the outcome for patients with PnCa remains poor and new therapeutic approaches with improved effectiveness and lower toxicity are needed.

Epidermal growth factor receptor (EGFR) overexpression is found in > 90% of cases of PnCa and represents an attractive target for the development of novel therapies (Troiani et al., 2012). However, clinical trials of anti-EGFR monoclonal antibodies, cetuximab (Erbitux; Eli Lilly) (Crane et al., 2011) or panitumumab (Vectibix; Amgen) (Halfdanarson et al., 2019) combined with chemotherapy have not been as encouraging as hoped, possibly due to downstream KRAS mutation in PnCa which obviates the effects of blocking EGFR (Eser et al., 2014). However, this does not preclude EGFR-targeted radioimmunotherapy (RIT) of PnCa, since EGFR overexpression is used only to selectively deliver radiation to tumours. Indeed, we recently reported that panitumumab modified with a metal-chelating polymer (MCP) to complex the β-particle emitter, ^177^Lu [Eβmax = 0.498 MeV (78.6%), 0.385 MeV (9.1%), 0.176 MeV (12.2%); t_1/2_ = 6.7 days] inhibited the growth of subcutaneous (s.c.) PANC-1 human PnCa xenografts in NRG (NOD-Rag1^−/−^ IL2Rg^null^) mice despite these tumours exhibiting KRAS mutation (Ma et al., 2013), while treatment with unlabeled panitumumab was ineffective (Aghevlian et al., 2019). The β-particles emitted by ^177^Lu have a maximum 2 mm range in tissues, which results in low linear energy transfer (LET < 0.3 keV/μm). Our group has also been exploring RIT of tumours exploiting the very low energy (< 20 keV) but high LET (LET = 4–26 keV/μm) Auger electrons (AEs) emitted by ^111^In (Ku et al., 2019). These nanometer-micrometer range electrons cause lethal DNA double-strand breaks (DSB) in cancer cells, especially if emitted in close proximity to the cell nucleus. The potency of [^111^In]In-labeled radioimmunoconjugates (RICs) is amplified by modification with nuclear translocation sequence (NLS) peptides which transport the RICs to the cell nucleus (Costantini et al., 2007). Interestingly, the EGFR harbours an endogenous NLS in the transmembrane domain that mediates nuclear importation of EGF (Lo et al., 2006) and cetuximab (Liao and Carpenter, 2009). Thus modification of RICs with an exogenous NLS may not be required for EGFR-targeted RIT. Moreover, recent studies have shown that a local bystander effect extends the therapeutic effects of AEs beyond the physical range of the electrons (Paillas et al., 2016). In addition, ^111^In emits two abundant γ-photons [Eγ-171 keV (90%) and 245 keV (94%)] and ^177^Lu emits a low abundance γ-photon [Eγ = 208 keV (11%)] which enable single photon emission computed tomography (SPECT), providing an opportunity to combine tumour imaging and RIT (i.e. “theranostic” concept). We previously proposed that panitumumab-MCP dual-labeled with ^177^Lu and ^111^In could represent a novel theranostic agent that combines SPECT and AE and β-particle mediated RIT (Aghevlian et al., 2018).

In the current study, we hypothesized that panitumumab labeled with AE-emitting, ^111^In or β-particle emitting, ^177^Lu would be effective for RIT of s.c. PANC-1 human PnCa xenografts in non-obese diabetic severe combined immunodeficiency (NOD/SCID) or NRG mice, respectively, at administered amounts that are non-toxic to normal tissues. Panitumumab was modified with DOTA (1,4,7,10-tetraazacyclododecane-1,4,7,10-tetraacetic acid) or conjugated to a MCP harbouring multiple DOTA to complex ^111^In or ^177^Lu. We previously reported that conjugation of panitumumab to this same MCP enables high specific activity (SA) labeling with ^111^In or ^177^Lu (Aghevlian et al., 2018). RIT of s.c. PANC-1 tumours in NRG mice with panitumumab-MCP-^177^Lu was effective at administered amounts that were non-toxic to normal tissues (Aghevlian et al., 2019).

## Methods

### Cell lines and tumour xenografts

EGFR positive PANC-1 human PnCa cells (4.0 × 10^5^ EGFR/ cell) (Korc et al., 1986) were purchased from the American Type Culture Collection (ATCC) and cultured in Dulbecco’s Modified Eagle’s Medium (DMEM) with high glucose (Gibco) supplemented with 1% penicillin and streptomycin and 10% fetal bovine serum (Gibco-Invitrogen). Tumour xenografts (7–9 mm diameter) were established at 4 weeks post s.c. inoculation of 3–4 × 10^6^ PANC-1 cells in DMEM (100 μL) into the left flank in female NOD/SCID mice (Charles River) or in NRG mice (University Health Network, Toronto, ON). All animal studies were conducted in compliance with the Canadian Council on Animal Care (CCAC) guidelines under a protocol (AUP 2843.3) approved by the institutional Animal Care Committee at the University Health Network.

### Radioimmunoconjugates (RICs)

The synthesis of hydrazino nicotinamide (HyNic) end-capped metal-chelation polymers (HyNic-MCP; M_r_ = ~ 33 kDa) that harbour 13 DOTA and 10 pendant polyethylene glycol (PEG) chains was previously reported (Lu et al., 2017). Panitumumab-MCP (M_r_ = ~ 220 kDa) was constructed by conjugating panitumumab IgG (Vectibix, Amgen) to an average of two HyNic-MCP through a 4-formylbenzamide moiety introduced into the IgG (Aghevlian et al., 2018). The synthesis and characterization of DOTA-conjugated panitumumab was previously reported (Aghevlian et al., 2018). The immunoconjugates were purified and exchanged into 0.1 M 4-(2-hydroxyethyl)-1-piperazineethanesulfonic acid (HEPES) buffer pH 5.0 by ultrafiltration on an Amicon spin filter (30 kDa MWCO; Millipore) repeated 6 times. The purified immunoconjugates (5–70 μg; 4–12 μL) were labeled with 0.75–49 MBq of [^111^In]InCl_3_ (Nordion) or [^177^Lu]LuCl_3_ (PerkinElmer) at 42 °C for 2 h (Aghevlian et al., 2018). ^111^In was no carrier added, radionuclide purity > 99.9% and activity concentration > 0.925 MBq/μL. ^177^Lu had a specific activity > 370 GBq/mg, radionuclide purity > 99% and activity concentration > 3.7 MBq/μL. The final radiochemical purity was > 95% measured by instant thin-layer silica gel chromatography (ITLC-SG; Pall) in 0.1 M sodium citrate, pH 5.5 or by size-exclusion HPLC (SE-HPLC) on a BioSep SEC-S2000 column (Phenomenex) eluted with 100 mM NaH_2_PO_4_ buffer, pH 7.0 at a flow rate of 0.8 mL/min with UV (280 nm) and radioactivity detection. The EGFR binding affinity was measured in a saturation binding assay using MDA-MB-468 human breast cancer cells (1.3 × 10^6^ EGFR/cell) (Aghevlian et al., 2018). The dissociation constant (K_d_) for binding of panitumumab-MCP-[^177^Lu]Lu and panitumumab-DOTA-[^177^Lu]Lu to EGFR on these cells was 2.2 ± 0.6 nmol/L and 1.0 ± 0.4 nmol/L, respectively (Aghevlian et al., 2018). The K_d_ values of [^111^In]In-labeled RICs were not measured since substitution of ^111^In for ^177^Lu was not expected to change the K_d_.

### Clonogenic survival assays and measurement of DNA DSBs

Clonogenic survival assays were performed by exposing 2 × 10^5^ PANC-1 cells in 2.0 mL of growth medium in 24-well plates to 2.5 nmoles/L (0.3, 0.6 or 1.2 MBq) of panitumumab-DOTA-[^177^Lu]Lu, panitumumab-DOTA-[^111^In]In or panitumumab-MCP-[^111^In]In for 16 h at 37 °C in growth medium or to unlabeled panitumumab-DOTA or medium alone. Approximately 700 cells were then seeded into 6-well plates and cultured for 12 days. Surviving colonies were stained with methylene blue and colonies (> 50 cells) counted. The plating efficiency (PE) was determined by dividing the number of colonies formed by the number of cells seeded. The surviving fraction (SF) was calculated by dividing the PE for treated cells by that for untreated cells. Unrepaired DNA double-strand breaks (DSBs) were assessed in the nucleus of PANC-1 cells by immunofluorescence for phosphorylated gamma histone-2A (γ-H2AX) as previously described (Cai et al., 2008). The images were analyzed for the integrated density of γ-H2AX foci per nucleus area using ImageJ software (Cai et al., 2009).

### Subcellular fractionation and cellular dosimetry

The subcellular distribution of activity on the cell membrane (CM), in the cytoplasm (Cy) or in the nucleus (N) of PANC-1 cells incubated with panitumumab-DOTA-[^177^Lu]Lu were measured to estimate the absorbed doses in the nucleus for panitumumab-DOTA-[^177^Lu]Lu and panitumumab-DOTA-[^111^In]In. ^111^In and ^177^Lu are both strongly bound by DOTA [log K_M_ = 25.4 and 23.9–24.5, respectively] (Baranyai et al., 2020) thus, it was assumed that the subcellular localization of panitumumab-DOTA-[^111^In]In was predicted by that of panitumumab-DOTA-[^177^Lu]Lu. Briefly, 2 × 10^5^ cells cultured overnight in wells in a 24-well plate were incubated for 1, 4, 8, or 24 h at 37 °C with panitumumab-DOTA*-*[^177^Lu]Lu (1.2 MBq; 240 MBq/nmole; 2.5 nmoles/L). Incubation was performed in the absence or presence of excess panitumumab, or with non-specific IgG-DOTA-[^177^Lu]Lu to assess EGFR-mediated cellular uptake. The medium was removed and the cells rinsed with phosphate-buffered saline (PBS), pH 7.5. Activity on the CM was displaced with 1 mL of 200 mM sodium acetate/500 mM NaCl, pH 2.5 at 22 °C. This procedure was repeated twice and the combined CM fractions measured in a γ-counter. The cells were then lysed with 2 × 500 μL of Nuclei EZ Lysis buffer (Sigma-Aldrich) on ice for 60 min. Lysed cells were transferred to 1.5 mL Eppendorf tubes and the tubes centrifuged at 3000×g for 5 min to separate the N and Cy activity (supernatant), which were measured in a γ-counter. The subcellular distribution of panitumumab-MCP-[^177^Lu]Lu in PANC-1 cells was previously reported (Aghevlian et al., 2019) and was used to predict the subcellular localization of panitumumab-MCP-[^111^In]In .

The absorbed dose in the nucleus of a PANC-1 cell over the 16 h incubation period used for clonogenic survival assays with panitumumab-DOTA-^177^Lu, panitumumab-DOTA-^111^In or panitumumab-MCP-^111^In, were estimated as $\sum {D}_{T\leftarrow S}=\sum {\overset{\sim }{A}}_S\times {S}_{T\leftarrow S}$, where *D*_*T* ← *S*_ is the mean dose in the nucleus (Gy) from activity in a subcellular or extracellular source compartment (CM, Cy, N or medium), ${\overset{\sim }{A}}_S$ is the time-integrated activity (Bq × sec) in the source compartment, and S is the Snyder factor (Goddu et al., 1994). ${\overset{\sim }{A}}_{S\ 0-16h}$ in each cell compartment or medium was calculated from the area under the curve from 0 to 16 h. Monte Carlo (MCNP Ver. 5.0; Los Alamos National Laboratory) was used to calculate the medium and monolayer S-values for ^177^Lu and ^111^In using the dimensions of a PANC-1 cell and N (radius = 8.5 μm and 7.0 μm; respectively) (Cai et al., 2017; Cai et al., 2010). The study volume was defined as a cylinder with a radius of 0.77 cm containing 1.05 cm thickness water and a 0.1 cm thick polystyrene bottom on which a monolayer of closely packed cells was attached. The detailed spectra of Auger electron, conversion electron, X-rays and γ-photons of ^111^In and ^177^Lu, as well as the β full energy spectrum of ^177^Lu were taken from the MIRD Radionuclide Data (Eckerman and Endo, 2008) and used in the MCNP5 simulation. MCNP5 uses continuous-energy nuclear and atomic data libraries with the energy cutoff for both electrons and photons at 1 keV. 2 × 10^6^ electrons or photons were launched for each calculation on the energy deposition per starting particle per tally volume to reach a statistical relative error lower than 0.05 of one standard deviation. We were not able to estimate the absorbed doses in the nucleus resulting from the 10 days in culture used for the clonogenic survival assays, since we did not fractionate the cells during this period, but estimation of the doses for the 16 h incubation allowed correlation of the effects of absorbed dose with SF after exposure to the RICs. In order to appreciate the relative biological effectiveness (RBE) of the AEs emitted by ^111^In compared to β-particles emitted by ^177^Lu, the SF was plotted vs. absorbed dose (Gy) for PANC-1 cells exposed to panitumumab-MCP-[^111^In]In, panitumumab-DOTA-[^111^In]In or panitumumab-DOTA-[^177^Lu]Lu.

### Normal tissue toxicity

Normal tissue toxicity was assessed in non-tumour bearing NOD/SCID mice (*n* = 5) injected i.v. (tail vein) with 10.0 MBq (10 μg; ~ 0.07 nmoles) of panitumumab-DOTA*-*[^111^In]In or panitumumab-MCP*-*[^111^In]In. This amount was selected because Ochakovskaya et al. (Ochakovskaya et al., 2001) found that ^111^In-labeled anti-CD74 antibodies administered up to 12.9 MBq in SCID mice caused no toxicity. Since NOD/SCID mice, similar to SCID mice harbor a germ line mutation in DNA repair (Biedermann et al., 1991) and panitumumab-DOTA-[^177^Lu]Lu may be more toxic than panitumumab-DOTA-[^111^In]In, due to the cross-fire effect of the moderate energy β-particles (Reilly, 2005), we assessed the normal tissue toxicity of panitumumab-DOTA-[^177^Lu]Lu at a lower activity (6.0 MBq; 10 μg; ~ 0.07 nmoles) in non-tumour bearing NRG mice which are not deficient in DNA repair. Control NOD/SCID or NRG mice received normal saline or panitumumab (10 μg; ~ 0.07 nmoles). Body weight was monitored every 2–4 days for 14 days. The 14 days period for assessment of toxicity was selected based on the requirements of Health Canada for acute toxicity testing of new therapeutic agents (Anonymous, 1995). At 14 days, the mice were sacrificed, and blood samples were collected for measurement of alanine aminotransferase (ALT) and creatinine (Cr). A complete blood cell (CBC) count and hemoglobin (Hb) were also obtained on a HemaVet 950FS instrument (Drew Scientific).

### Radioimmunotherapy (RIT) studies

**Scheme 1:**
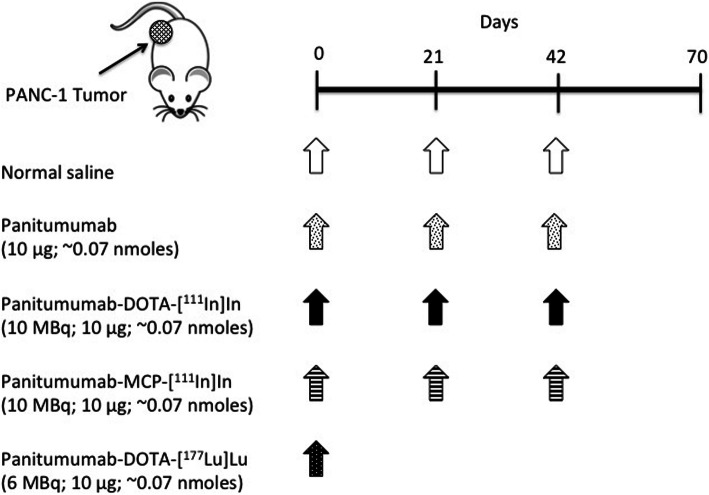
Design of radioimmunotherapy (RIT) studies. NOD/SCID or NRG mice were implanted s.c. with EGFR-positive PANC-1 human pancreatic cancer xenografts. Tumour-bearing NOD/SCID mice were treated with fractionated amounts of panitumumab (10 μg; ~ 0.07 nmoles), panitumumab-DOTA-[^111^In]In (10 MBq; 10 μg; ~ 0.07 nmoles) or panitumumab-MCP-[^111^In]In (10 MBq; 10 μg; ~ 0.07 nmoles) or normal saline every 3 weeks for 3 cycles. Tumour-bearing NRG mice were treated with a single amount of panitumumab-DOTA-[^177^Lu]Lu (6 MBq; 10 μg; ~ 0.07 nmoles) or panitumumab (10 μg; ~ 0.07 nmoles) or normal saline.

NOD/SCID mice with s.c. PANC-1 tumours were treated with 10 MBq (10 μg) of panitumumab-DOTA-[^111^In]In (*n* = 12) or panitumumab-MCP-[^111^In]In (*n* = 7) administered i.v. (tail vein) in 100 μL of normal saline every 3 weeks for 9 weeks (Scheme 1). NRG mice with s.c. PANC-1 tumours were injected i.v. with a single amount of 6 MBq (10 μg; ~ 0.07 nmoles) of panitumumab-DOTA-[^177^Lu]Lu in 100 μL of normal saline. Control NOD/SCID or NRG mice received panitumumab (10 μg; *n* = 10) or normal saline (n = 10). Tumour length (mm) and width (mm) were measured using calipers every 2–3 days until the humane endpoint was reached. The tumour volume (V = mm^3^) was calculated as V = length × (width)^2^ × 0.5 (Euhus et al., 1986). The tumour growth index (TGI) was calculated by dividing the tumour volume at each observation time point by the volume at the start of treatment. Similarly, the body weight index (BWI) was calculated by dividing the body weight at each time point by the pre-treatment body weight. The TGI vs. time curves were fitted to an exponential growth model by GraphPad Prism Ver. 4.0 and the tumour doubling time (TDT) was calculated.

### Tumour and normal organ dosimetry

Biodistribution studies were performed in NOD/SCID or NRG mice with s.c. PANC-1 tumours injected i.v. (tail vein) with panitumumab-DOTA-[^111^In]In (10 MBq; 10 μg) or panitumumab-DOTA-[^177^Lu]Lu (6 MBq; 10 μg) to estimate the absorbed doses in the tumour and normal organs. Briefly, at selected times from 24 to 168 h p.i., groups of mice (*n* = 5) were sacrificed, and the tumour and normal organs collected, weighed and measured in a γ-counter. The tumour and normal organ biodistribution of panitumumab-MCP-[^177^Lu]Lu was previously reported (Aghevlian et al., 2019). The doses were calculated as $\sum {D}_{T\leftarrow S}=\sum {\overset{\sim }{A}}_S\times {S}_{T\leftarrow S}$, where *D*_*T* ← *S*_ is the mean absorbed dose in the target organ (Gy) from activity in a source organ, ${\overset{\sim }{A}}_S$ is the time-integrated activity (Bq × sec) in the source organ, and S is the Snyder factor. For the tumour, ${\overset{\sim }{A}}_{0-168h}$ was obtained from the AUC_0-168h_ of a plot of activity (Bq) vs. time (secs). Only for the tumour, ${\overset{\sim }{A}}_{168-\infty }$ was calculated by dividing the activity at 168 h p.i. of the RICs by the decay constants for ^111^In (2.9 × 10^− 6^ s^−1^) or ^177^Lu (1.2 × 10^− 6^ s^−1^), thus assuming elimination after the final time point solely by radioactive decay, since further biological elimination was not anticipated in the tumour. For normal organs, the uptake phase, ${\overset{\sim }{A}}_{0-24h}$ was calculated by the Trapezoidal Rule as 0.5 × 24 h × 3600 s/hours × the activity (Bq) at 24 h. The elimination phase, ${\overset{\sim }{A}}_{24-\infty }$ was calculated by the AUC obtained by fitting a plot of activity (Bq) vs. time (secs) to a single exponential elimination curve. ${\overset{\sim }{A}}_{0-\infty }$ was calculated as the sum of ${\overset{\sim }{A}}_{0-168h}$ and ${\overset{\sim }{A}}_{168-\infty }$ for the tumour and ${\overset{\sim }{A}}_{0-24h}$ and ${\overset{\sim }{A}}_{24-\infty }$ for normal organs. S-values for mouse organs were used (Bitar et al., 2007). The tumour dose was estimated using the sphere model in OLINDA/EXM radiation dosimetry software (Stabin et al., 2005).

### Statistical analysis

Results were expressed as mean ± SD. Statistical significance was tested using an unpaired two-tailed Student’s t-test or one-way ANOVA (*P* < 0.05).

## Results

### Clonogenic survival assays and measurement of DNA DSBs

**Fig. 1:**
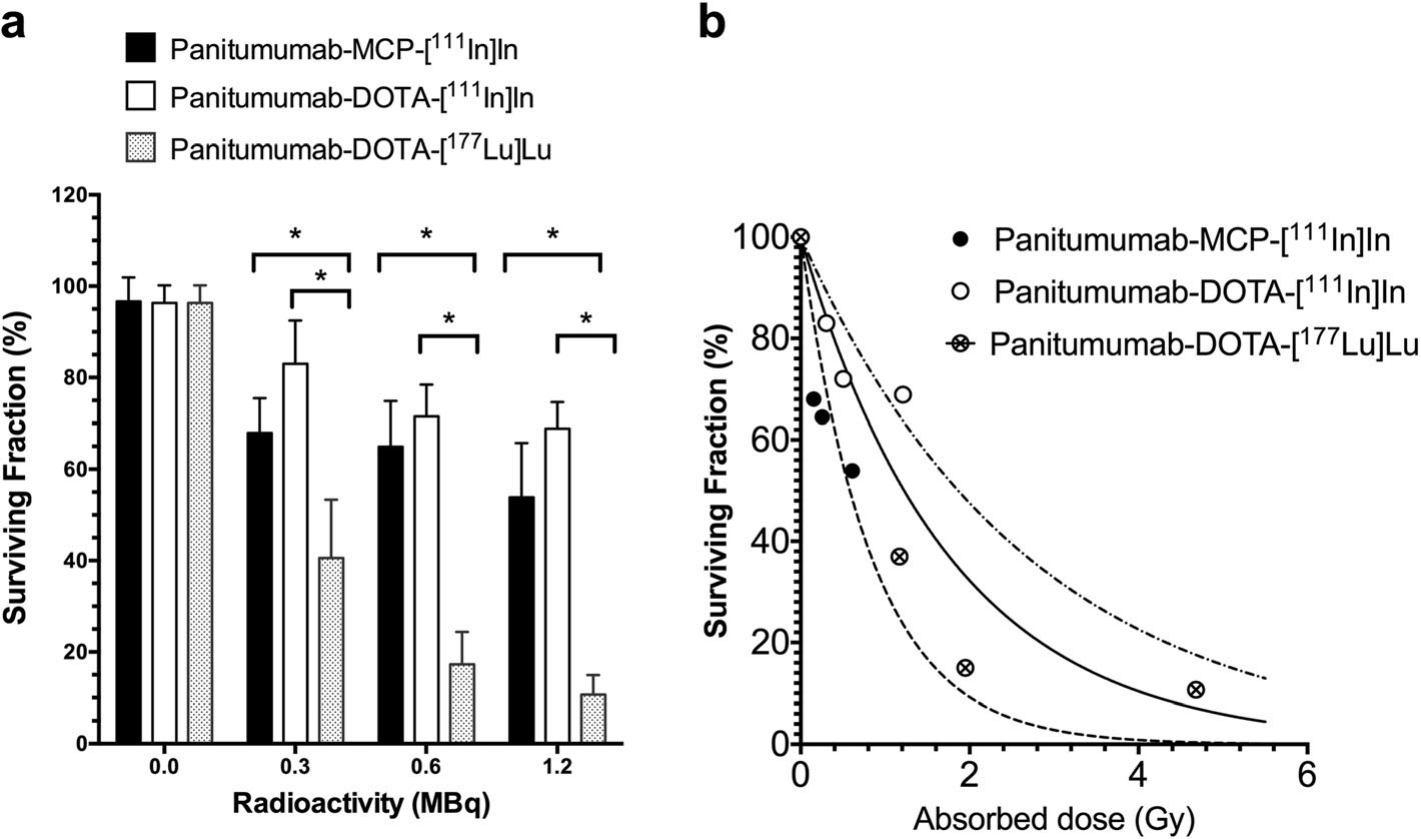
**a** Surviving fraction of human PANC-1 cells exposed to 0.3, 0.6 or 1.2 MBq (2.5 nmoles/L) of panitumumab-DOTA-[^111^In]In, panitumumab-MCP-[^111^In]In, or panitumumab-DOTA-[^177^Lu]Lu or unlabeled radioimunoconjugaes (RICs; 0.0 MBq) in 2.0 mL of growth medium. Values shown are the mean ± SD (*n* = 3). Significant differences (*P* < 0.05) are noted by asterisks. **b** Surviving fraction of PANC-1 cells exposed to RICs vs. absorbed dose (Gy) in the nucleus. Absorbed dose was calculated as described in the Methods and shown in Table 1. The data was fitted to a survival model: SF = exp.(−a*dose). Fitting revealed that a = 1.19 ± 0.23 for panitumumab-MCP-[^111^In]In; 0.37 ± 0.08 for panitumumab-DOTA-[^111^In] and 0.56 ± 0.11 for panitumumab-DOTA-[^177^Lu]Lu

Clonogenic survival and DNA DSB assays were performed at constant RIC concentration of 2.5 nM and increasing apparent molar activity from 60 to 240 MBq/nmole at the time of adding the RICs to the cells. There was no effect of unlabeled panitumumab-MCP or panitumumab-DOTA on the SF of PANC-1 cells (Fig. 1a; 0.0 MBq). Panitumumab-DOTA-[^177^Lu]Lu was significantly more cytotoxic than panitumumab-MCP-[^111^In]In or panitumumab-DOTA-[^111^In]In at all amounts tested (0.3–1.2 MBq; 0.005 nmoles in 2.0 mL of growth medium). At the maximum amount tested (1.2 MBq), panitumumab-DOTA-[^177^Lu]Lu reduced the SF of PANC-1 cells to 10.7 ± 2.5% vs. 53.9 ± 6.9% for panitumumab-MCP-[^111^In]In and 68.9 ± 3.3% for panitumumab-DOTA-[^111^In]In (*P* < 0.05). There were no significant differences in the SF of PANC-1 cells treated with panitumumab-MCP-[^111^In]In or panitumumab-DOTA-[^111^In]In at any amount tested. The absorbed doses in the nucleus were estimated for RICs incubated with PANC-1 cells (Fig. 1b and Table 1) based on subcellular fractionation studies and applying cellular dosimetry (see next section). The SF vs. dose was plotted and fitted to a survival model: SF = exp.(−a*dose). Fitting revealed that a = 1.19 ± 0.23 Gy^− 1^ for panitumumab-MCP-[^111^In]In; 0.37 ± 0.08 Gy^− 1^ for panitumumab-DOTA-[^111^In]In and 0.56 ± 0.11 Gy^− 1^ for panitumumab-DOTA-[^177^Lu]Lu. The cytotoxicity per Gy in the nucleus was highest for panitumumab-MCP-[^111^In]In, followed by panitumumab-DOTA-[^177^Lu]Lu, then panitumumab-DOTA-[^111^In]In, but there were limited data points to fit the curves for ^111^In-labeled RICs.

**Table 1:** Doses in the Nucleus of a PANC-1 Cell Deposited by RICs^a^

Source Compartment	S-Value (^111^In) (Gy/(Bq × sec)	Panitumumab-DOTA-[^111^In]In		Panitumumab-MCP-[^111^In]In		S-Value (^177^Lu) (Gy/(Bq × sec)	Panitumumab-DOTA-[^177^Lu]Lu	
		Ã_s_ (Bq × sec)	Dose (Gy)	Ã_s_ (Bq × sec)	Dose (Gy)		Ã_s_ (Bq × sec)	Dose (Gy)
CS	7.35 × 10^−5^	9.24 × 10^3^	0.68	2.67 × 10^3^	0.20	3.55 × 10^− 4^	9.77 × 10^3^	3.47
Cy	1.03 × 10^− 4^	1.01 × 10^3^	0.10	1.16 × 10^3^	0.12	4.02 × 10^− 4^	1.07 × 10^3^	0.43
N	5.06 × 10^− 4^	0.67 × 10^3^	0.34	0.40 × 10^3^	0.20	6.93 × 10^− 4^	0.70 × 10^3^	0.49
Total Ã_s_ (cell; Bq × sec)		10.92 × 10^3^		4.23 × 10^3^			11.54 × 10^3^	
Medium	1.50 × 10^−12^	5.91 × 10^10^	0.09	6.19 × 10^10^	0.09	4.72 × 10^− 12^	6.20 × 10^10^	0.29
Total dose (cell and medium)			1.21		0.61			4.68
Major Contributor			CS (56%)		CS/N (30%)			CS (74%)

^a^2 × 10^5^ PANC-1 cells were incubated with 1.2 MBq (2.5 nmoles/L) of RICs in 2.0 mL of growth medium for 16 h at 37 °C, then seeded and cultured for 10 days

**Fig. 2:**
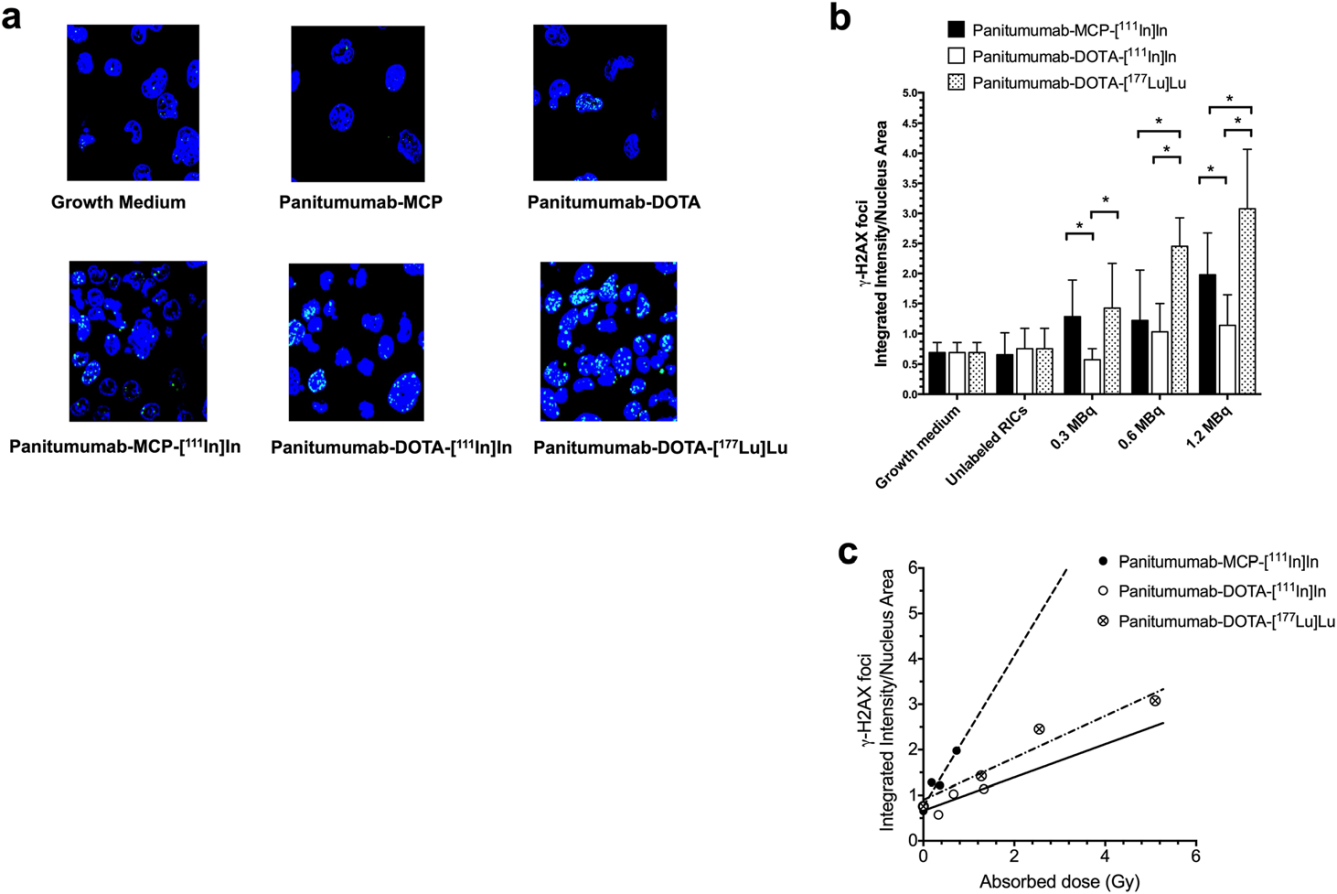
**a** Representative immunofluorescence microscopy images of PANC-1 cells exposed to growth medium, unlabeled panitumumab-MCP or panitumumab-DOTA or 1.2 MBq (2.5 nmoles/L) of panitumumab-MCP-[^111^In]In, panitumumab-DOTA-[^111^In]In or panitumumab-DOTA-[^177^Lu]Lu showing γ-H2AX foci in the nucleus, counterstained blue with DAPI. **b** Integrated density of γ-H2AX foci representing unrepaired DNA DSBs in the nucleus of PANC-1 cells exposed to RICs. Values shown are the mean ± SD (*n* = 12). Significant differences (*P* < 0.05) are noted by the asterisks. **c** Integrated density of γ-H2AX foci in the nucleus of PANC-1 cells exposed to RICs vs. absorbed dose (Gy) in the nucleus. Lines were obtained by linear regression

Cells exposed to growth medium or unlabeled panitumumab-MCP or panitumumab-DOTA exhibited few γ-H2AX foci, representing sites of unrepaired DNA DSBs (Fig. 2a) and the density of these foci were not significantly different than in untreated cells. Panitumumab-DOTA-[^177^Lu]Lu caused significantly more γ-H2AX foci in the nucleus of PANC-1 cells than panitumumab-DOTA-^111^In at all amounts (Fig. 2b) and more γ-H2AX foci than panitumumab-MCP-^111^In at 0.6 MBq and 1.2 MBq, but not at 0.3 MBq. Panitumumab-MCP-[^111^In]In caused more γ-H2AX foci than panitumumab-DOTA-[^111^In]In at 0.3 MBq and 1.2 MBq, but not at 0.6 MBq. The higher density of γ-H2AX foci for panitumumab-DOTA-[^177^Lu]Lu agreed with the higher absorbed dose in the nucleus of PANC-1 cells than panitumumab-MCP-[^111^In]In or panitumumab-DOTA-[^111^In]In (Table 1). The integrated density of γ-H2AX foci in the nucleus of PANC-1 cells exposed to RICs vs. absorbed dose to the nucleus (Gy) was fitted by linear regression (Fig. 2c). The slope for panitumumab-MCP-[^111^In]In (1.7 ± 0.4) was highest, followed by panitumumab-DOTA-[^177^Lu]Lu (0.46 ± 0.08) and panitumumab-DOTA-[^111^In]In (0.36 ± 0.19). Thus, both the clonogenic survival and DNA DSB assays suggested that on a per Gy basis, the cytotoxicity decreased in the order of panitumumab-MCP-[^111^In]In, panitumumab-DOTA-[^177^Lu]Lu and panitumumab-DOTA-[^111^In]In.

### Subcellular fractionation and cellular dosimetry

The percentage of activity bound by PANC-1 cells incubated with panitumumab-DOTA-[^177^Lu]Lu (2.5 nnmoles/L) increased over 24 h [Supplementary Information Fig. S1a]. Co-incubation with a 50-fold molar excess of panitumumab (125 nmoles/L) significantly decreased the binding of panitumumab-DOTA-[^177^Lu]Lu to PANC-1 cells, and the binding was similar to that for non-specific hIgG-DOTA-[^177^Lu]Lu. Subcellular fractionation revealed that in cells incubated with panitumumab-DOTA-[^177^Lu]Lu, most activity was located on the CM, with much lower amounts in the Cy or in the N (Fig. S1b). There were no significant differences in the subcellular distribution from 1 h to 24 h. When incubated with 1.2 MBq (0.005 nmoles) of RICs in 2.0 mL of growth medium for 16 h, the absorbed dose in the N was 3.9-fold and 7.7-fold higher for panitumumab-DOTA-[^177^Lu]Lu (4.68 Gy) than panitumumab-DOTA-[^111^In]In (1.21 Gy) or panitumumab-MCP-[^111^In]In (0.61 Gy), respectively (Table 1). The SF vs. absorbed doses in the nucleus for exposure of PANC-1 cells to 0.3 MBq, 0.5 MBq and 1.2 MBq of RICs are plotted in Fig. 1b (results discussed in previous section).

### Normal tissue toxicity

**Fig. 3:**
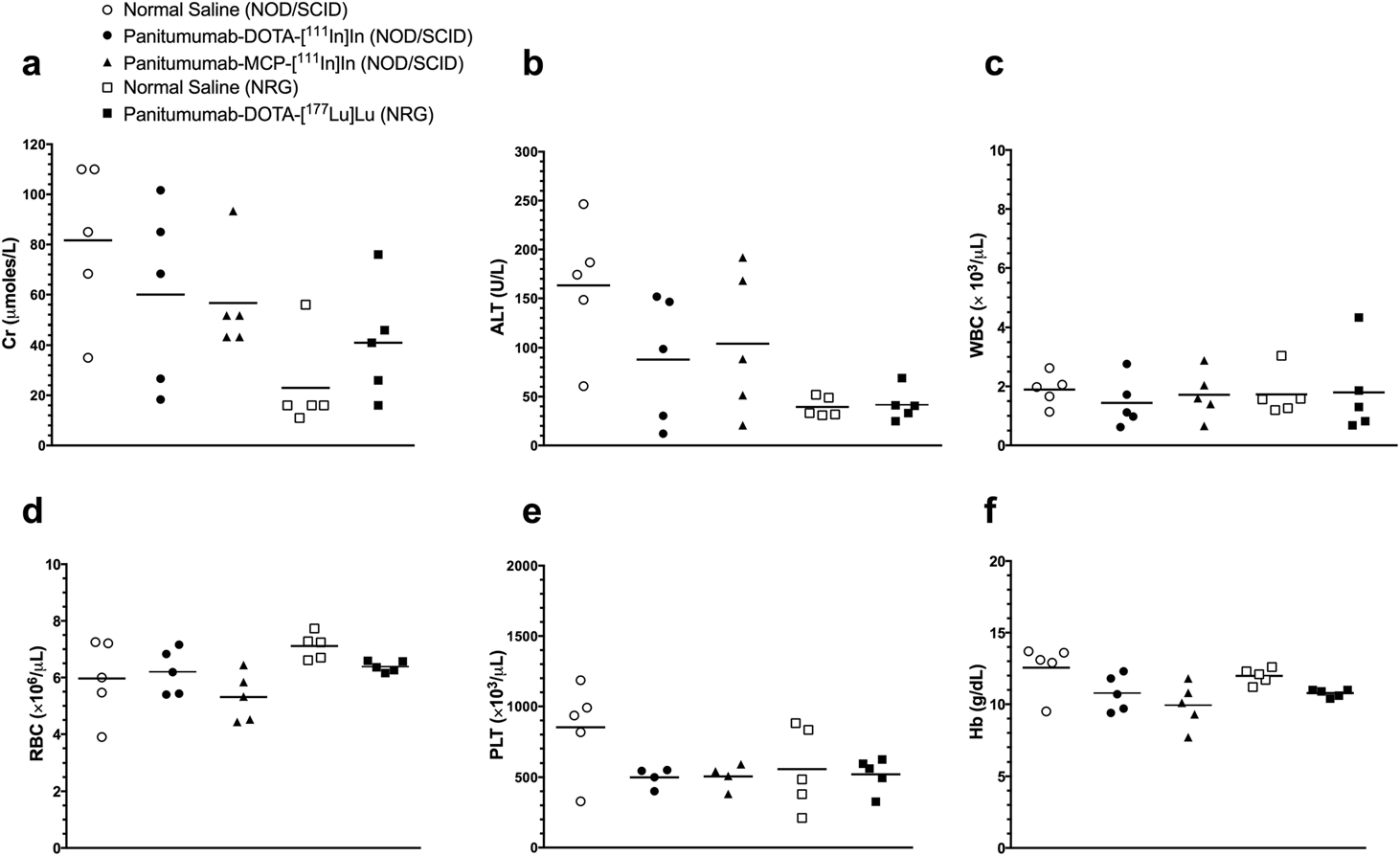
**a** Serum creatinine (Cr), **b** Alanine aminotransferase (ALT), **c** White blood cells (WBC), **d** Red blood cells (RBC), **e** Platelets (PLT), and (**f**) hemoglobin (Hb) in non-tumour bearing NOD/SCID mice at two weeks after i.v. injection (tail vein) of 10.0 MBq (10 μg; ~ 0.07 nmoles) of panitumumab-DOTA-[^111^In]In, panitumumab-MCP-[^111^In]In or normal saline, or in NRG mice injected i.v. with 6.0 MBq (10 μg; ~ 0.07 nmoles) of panitumumab-DOTA-[^177^Lu]Lu. Individual values and mean values (horizontal lines) are shown

Administration of 10.0 MBq (10 μg; ~ 0.07 nmoles) of panitumumab-DOTA-[^111^In]In or panitumumab-MCP-[^111^In]In or 6.0 MBq (10 μg; ~ 0.07 nmoles) of panitumumab-MCP-[^177^Lu]Lu to NOD/SCID or NRG mice, respectively, caused no significant increase in the mean serum Cr or ALT compared to mice receiving normal saline (Fig. 3a,b). Administration of these amounts of RICs caused no change in CBC or Hb (Fig. 3c-f). There were strain differences in normal Cr, ALT and CBC values between control NOD/SCID and NRG mice receiving normal saline.

### Radioimmunotherapy studies

**Fig. 4:**
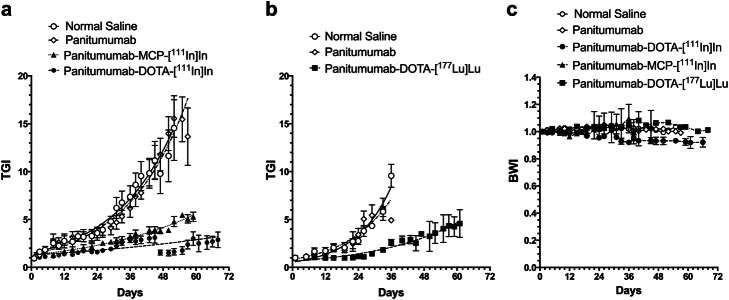
Tumour growth index (TGI) vs. time (days) in (**a**) NOD/SCID mice with s.c. PANC-1 xenografts treated with three i.v. (tail vein) injections (10 MBq; 10 μg; ~ 0.07 nmoles) of panitumumab-MCP-^111^In, panitumumab-DOTA-^111^In or unlabeled panitumumab (10 μg; ~ 0.07 nmoles) or normal saline separated by 3 weeks or in (**b**) NRG mice with s.c. PANC-1 xenografts treated with a single i.v. injection (10 MBq; 10 μg; ~ 0.07 nmoles) of panitumumab-DOTA-^177^Lu or unlabeled panitumumab (10 μg; ~ 0.07 nmoles) or normal saline. The TGI vs. time curves were fitted to an exponential growth model (lines) to estimate tumour doubling times. The body weight index (BWI) is shown in (**c**) for tumour-bearing NOD/SCID and NRG mice treated with the RICs, unlabeled panitumumab or normal saline

The mean tumour volumes prior to treatment of NOD/SCID mice with panitumumab-DOTA-[^111^In]In, panitumumab-MCP-[^111^In]In, unlabeled panitumumab or normal saline were 257 ± 170 mm^3^, 203 ± 65 mm^3^, 93 ± 38 mm^3^ and 104 ± 41 mm^3^. The TGI was defined as the tumour volume at the observation time point post-treatment divided by the initial tumour volume prior to treatment and was used to account for differences in the initial tumour volume between groups. Administration of three single amounts (10.0 MBq; 10 μg; ~ 0.07 nmoles) of panitumumab-DOTA-[^111^In]In or panitumumab-MCP-[^111^In]In separated by 3 weeks to NOD/SCID mice strongly inhibited PANC-1 tumour growth, while tumours grew rapidly in mice treated with unlabeled panitumumab (10 μg; ~ 0.07 nmoles) or normal saline (Fig. 4a). The TDT for treatment with panitumumab-DOTA-[^111^In]In, panitumumab-MCP-[^111^In]In, unlabeled panitumumab or normal saline were 51.8, 28.1, 15.3 and 15.6 days, respectively. At 43 days when at least 3 mice remained alive in each group, there was no significant difference in the mean ± SD TGI for RIT with panitumumab-DOTA-[^111^In]In or panitumumab-MCP-[^111^In]In (3.0 ± 1.5 vs. 3.9 ± 0.8, respectively; *P* > 0.05). However, RIT was 3.3-fold and 2.5-fold more effective, respectively than unlabeled panitumumab (TGI = 9.9 ± 4.1; *P* < 0.001) or normal saline (TGI = 9.8 ± 4.4; *P* < 0.001). Administration of a single amount (6.0 MBq; 10 μg; ~ 0.07 nmoles) of panitumumab-DOTA-[^177^Lu]Lu to NRG mice significantly inhibited PANC-1 tumour growth compared to unlabeled panitumumab or normal saline (Fig. 4b). The TDT were 20.9, 11.5 and 9.1 days for treatment with panitumumab-DOTA-[^177^Lu]Lu, unlabeled panitumumab and normal saline, respectively. At 33 days, when at least 3 mice remained alive in each group, the mean ± SD TGI for NRG mice treated with panitumumab-DOTA-[^177^Lu]Lu (1.8 ± 0.7) was significantly lower than in NOD/SCID mice receiving unlabeled panitumumab or normal saline (6.1 ± 2.0 and 5.8 ± 0.9, respectively; *P* < 0.001). There were no significant decreases in BWI for NOD/SCID or NRG mice treated with RICs compared to control mice receiving unlabeled panitumumab or normal saline (Fig. 4c).

### Tumour and normal organ dosimetry

The absorbed doses in s.c. PANC-1 tumours after administration of three amounts (10 MBq; 10 μg; ~ 0.07 nmoles) of panitumumab-DOTA-[^111^In]In or panitumumab-MCP-[^111^In]In to NOD/SCID mice were 8.8 ± 3.0 Gy and 2.6 ± 0.3 Gy, respectively (Table 2; *P* < 0.006). The absorbed dose in the tumour after injection of a single amount (6.0 MBq; 10 μg) of panitumumab-DOTA-[^177^Lu]Lu to NRG mice with s.c. PANC-1 xenografts was 11.6 ± 4.9 Gy, which was not significantly different than that for panitumumab-DOTA-[^111^In]In (*P* = 0.367) but was 4.5-fold significantly higher than panitumumab-MCP-[^111^In]In (*P* = 0.01). The highest absorbed doses in normal organs were in the liver, spleen and kidneys for all RICs and in the pancreas for panitumumab-DOTA-[^177^Lu]Lu. The absorbed dose in the kidneys was significantly lower for panitumumab-DOTA-[^111^In]In (5.3 ± 1.7 Gy; *P* = 0.0485) and panitumumab-MCP-[^111^In]In (5.2 ± 0.3 Gy; *P* = 0.0038) than panitumumab-DOTA-[^177^Lu]Lu (7.8 ± 1.1 Gy). The liver and spleen doses for panitumumab-MCP-[^111^In]In (6.1 ± 0.6 Gy and 7.5 ± 0.7 Gy) were 2.2-fold and 1.7-fold significantly higher, respectively, than panitumumab-DOTA-[^111^In]In (2.8 ± 0.6 Gy; *P* = 0.0002 and 4.5 ± 1.4 Gy; *P* = 0.0086) due to greater uptake of panitumumab-MCP-[^111^In]In in these organs (Supplementary Information Fig. S2). The whole body absorbed doses for administration of panitumumab-DOTA-[^177^Lu]Lu (1.3 ± 0.1 Gy) was significantly lower than panitumumab-DOTA-[^111^In]In (1.7 ± 0.2 Gy, *P* = 0.0117), but was not significantly different from panitumumab-MCP-[^111^In]In (1.5 ± 0.1 Gy, *P* = 0.207).

**Table 2:** Absorbed doses in the tumour and normal organs in mice with subcutaneous PANC-1 xenografts treated with RICs

	Absorbed Dose (Mean ± SD; Gy) ^a^		
Organ	Panitumumab-DOTA-[^111^In]In ^b^	Panitumumab-MCP-[^111^In]In ^b^	Panitumumab-DOTA-[^177^Lu]Lu ^c^
Heart	1.9 ± 0.4	2.2 ± 0.1	2.5 ± 0.3
Lungs	2.5 ± 0.7	2.3 ± 0.3	4.0 ± 1.3
Liver	2.8 ± 0.6	6.1 ± 0.6	6.5 ± 0.9
Spleen	4.5 ± 1.4	7.5 ± 0.7	6.3 ± 1.1
Pancreas	1.4 ± 0.2	2.0 ± 0.3	5.3 ± 1.9
Stomach	1.2 ± 0.2	1.8 ± 0.1	0.9 ± 0.1
Intestine	1.5 ± 0.3	1.4 ± 0.2	0.6 ± 0.1
Kidneys	5.3 ± 0.7	5.2 ± 0.3	7.8 ± 1.1
Whole Body	1.7 ± 0.2	1.5 ± 0.1	1.3 ± 0.1
Tumour	8.8 ± 3.0	2.6 ± 0.3	11.6 ± 4.9

^a^Estimated using $D=\sum {\overset{\sim }{A}}_S\times {S}_{T\leftarrow S}$, where ${\overset{\sim }{A}}_S$ is the time-integrated activity (Bq × sec) in the source organ (see Supplementary Fig. S2), and S is the Snyder factor for mouse organs (Bitar et al., 2007). The tumour dose was estimated using the sphere model in OLINDA/EXM dosimetry software and the tumour size (Stabin et al., 2005)

^b^Absorbed dose for NOD/SCID mice injected i.v. (tail vein) with three amounts (10 MBq; 10 μg; ~ 0.07 nmoles) of ^111^In-labeled RICs separated by 3 weeks

^c^Absorbed dose for NRG mice injected i.v. (tail vein) with a single amount (6 MBq; 10 μg; ~ 0.07 nmoles) of ^177^Lu-labeled RICs

## Discussion

In this study, we evaluated the cytotoxicity in vitro of panitumumab labeled with AE-emitting, ^111^In or β-particle-emitting, ^177^Lu on PANC-1 human PnCa cells and correlated the SF of these cells in clonogenic assays with unrepaired DNA DSBs in the nucleus assessed by immunofluorescence for γ-H2AX as well as the absorbed dose in the nucleus. We further studied the effectiveness of ^111^In- and ^177^Lu-labeled panitumumab for treatment of s.c. PANC-1 tumours in vivo in NOD/SCID or NRG mice, respectively. Panitumumab was conjugated to DOTA chelators or to a MCP with multiple DOTA to complex ^111^In or to DOTA to complex ^177^Lu. Tumour-bearing mice were treated with the RICs at amounts that caused no normal tissue toxicity.

Panitumumab-DOTA-[^177^Lu]Lu was more potent in vitro for killing PANC-1 cells on a per MBq exposure basis than panitumumab-DOTA-[^111^In]In or panitumumab-MCP-[^111^In]In (Fig. 1a). However, comparing the surviving fraction of PANC-1 cells vs. absorbed dose in the nucleus (Gy) using the limited data points revealed that panitumumab-MCP-[^111^In]In was the most cytotoxic in vitro per Gy followed by panitumumab-DOTA-[^177^Lu]Lu, then panitumumab-DOTA-[^111^In]In (Fig. 1b). Nonetheless, panitumumab-DOTA-[^177^Lu]Lu caused a higher integrated density of DNA DSBs in the cell nucleus per MBq exposure (Fig. 2a,b and Table 1). Comparing DNA DSBs vs. absorbed dose indicated that panitumumab-MCP-[^111^In]In caused more DNA DSBs per Gy than panitumumab-DOTA-[^177^Lu]Lu which caused moderately more DSBs than panitumumab-DOTA-[^111^In]In (Fig. 2c).

The greater cyotoxicity of panitumumab-DOTA-[^177^Lu]Lu may be explained by the higher monolayer S-values to the nucleus for ^177^Lu than ^111^In (Table 1) which is contributed by cell-bound ^177^Lu and the cross-dose from neighbouring cells that also bound ^177^Lu. In addition, the cross-dose from ^177^Lu from activity in the medium during the 16 h incubation period used for the clonogenic assays may kill PANC-1 cells. However, cellular dosimetry revealed that 94% of the absorbed dose in the nucleus (4.39 Gy) was contributed by panitumumab-DOTA-[^177^Lu]Lu that was cell-bound or from the cross-dose from neighbouring cells that bound ^177^Lu with 74% of the dose due to decay of ^177^Lu on the CM of PANC-1 cells (3.47 Gy). The absorbed dose in the nucleus of PANC-1 cells exposed to panitumumab-MCP-[^111^In]In was 2.0-fold lower than panitumumab-DOTA-[^111^In]In, due to a 2.6-fold lower time-integrated cell-bound activity (total Ã_s_ = 4.2 × 10^3^ vs. 10.9 × 10^3^ Bq × sec; Table 1). The reason for the decreased uptake of panitumumab-MCP-[^111^In]In by PANC-1 cells is not known but may be due to slower kinetics of EGFR binding and internalization caused by the MCP modification. However, we previously found that panitumumab-MCP-[^177^Lu]Lu and panitumumab-DOTA-[^177^Lu]Lu exhibited similar EGFR binding affinities (K_d_ = 2.2 ± 0.6 nmol/L and 1.0 ± 0.4 nmol/L, respectively) (Aghevlian et al., 2018).

To select the amount of RICs to be administered for the RIT studies, we assessed the normal tissue toxicity of a single amount of 10.0 MBq (10 μg; ~ 0.07 nmoles) of panitumumab-DOTA-[^111^In]In or panitumumab-MCP-[^111^In]In administered to non tumour-bearing NOD/SCID mice or 6.0 MBq (10 μg; ~ 0.07 nmoles) of panitumumab-DOTA-[^177^Lu]Lu to NRG mice. These amounts caused no hematopoietic system, liver or kidney toxicity (Fig. 3). We previously reported no normal tissue toxicity for administration of 6.0 MBq (10 μg; ~ 0.07 nmoles) of panitumumab-MCP-[^177^Lu]Lu to NRG mice (Aghevlian et al., 2019). Liu et al. (Liu et al., 2014) found that RIT of Balb/c nude mice implanted with s.c. UM-SCC-22B head and neck squamous cell carcinoma (HNSCC) xenografts with 14.8 MBq of panitumumab-DOTA-^177^Lu caused no decrease in body weight, indicating no general normal tissue toxicity. Weber et al. (Weber et al., 2016) found that 8.0 MBq of ^177^Lu-labeled anti-CD22 antibodies administered to NRG mice similarly caused no decrease in body weight. Although the amounts of panitumumab-DOTA-[^111^In]In, panitumumab-MCP-[^111^In]In and panitumumab-DOTA-[^177^Lu]Lu administered to NOD/SCID or NRG mice in our study did not cause normal tissue toxicity, panitumumab does not bind to murine EGFR (Tabrizi et al., 2006). Thus EGFR-specific radiotoxicities caused by these RICs would need to be studied in humans in a future Phase 1 clinical trial. NOD/SCID mice harbor a germ line mutation in DNA repair and are radiosensitive (Biedermann et al., 1991) whereas NRG mice do not exhibit unusual radiosensitivity, Therefore, non-EGFR mediated normal tissue radiotoxicities from the RICs in these mice may be more predictive of those in humans.

Administration of three fractionated amounts (total = 30.0 MBq; 30 μg; ~ 0.21 nmoles) of panitumumab-MCP-[^111^In]In or panitumumab-DOTA-[^111^In]In separated by 3 weeks to NOD/SCID mice with s.c. PANC-1 xenografts (Scheme 1) strongly inhibited tumour growth, while three amounts of unlabeled panitumumab (total 30 μg; ~ 0.21 nmoles) had no effect on tumour growth (Fig. 4a). A single amount (6.0 MBq; 10 μg; ~ 0.07 nmoles) of panitumumab-DOTA-[^177^Lu]Lu (Scheme 1) inhibited PANC-1 tumour growth in NRG mice, while tumours in NRG mice treated with unlabeled panitumumab exhibited rapid growth (Fig. 4b). There was no decrease in body weight in mice receiving either ^111^In- or ^177^Lu-labeled RICs (Fig. 4c). Liu et al. (Liu et al., 2014) found that RIT with panitumumab-DOTA-[^177^Lu]Lu (14.8 MBq) arrested tumour growth in Balb/c nude mice with s.c. EGFR-positive UM-SCC-22B human HNSCC xenografts, while unlabeled panitumumab was not effective. In addition, we previously reported that 6.0 MBq (10 μg; ~ 0.07 nmoles) of panitumumab-MCP-[^177^Lu]Lu administered to NRG mce with s.c. PANC-1 tumours strongly inhibited tumour growth while unlabeled panitumumab was ineffective (Aghevlian et al., 2019).

The absorbed dose in PANC-1 tumours in NOD/SCID mice after administration of three amounts of panitumumab-DOTA-[^111^In]In (total = 30.0 MBq; 30 μg; ~ 0.21 nmoles) was 8.8 ± 3.0 Gy (Table 2). There was a lower absorbed dose in NOD/SCID mice treated with panitumumab-MCP-[^111^In]In (2.6 ± 0.3 Gy; Table 2), due to a lower time-integrated activity (Ã_s_) in the tumour (Fig. S2). The absorbed dose in the tumour from administration of a total of 30.0 MBq (30 μg; ~ 0.21 nmoles) of panitumumab-DOTA-[^111^In]In to NOD/SCID mice was similar to that for RIT with a single amount (6.0 MBq; 10 μg) of panitumumab-DOTA-[^177^Lu]Lu (11.6 ± 4.9 Gy) in NRG mice, while the tumour doses were 4.5-fold lower for panitumumab-MCP-[^111^In]In (Table 2). We previously reported that the absorbed dose in s.c. PANC-1 tumours in NRG mice administered 6.0 MBq (10 μg; ~ 0.07 nmoles) of panitumumab-MCP-^177^Lu was 12.3 ± 0.9 Gy (Aghevlian et al., 2019). MCP conjugation of panitumumab enables higher SA labeling with ^111^In or ^177^Lu than DOTA-panitumumab (Aghevlian et al., 2018) which could theoretically deliver more activity to tumour cells per EGFR binding event, resulting in a higher absorbed dose, but in the current study panitumumab-DOTA-[^111^In]In and panitumumab-MCP-[^111^In]In were labeled at the same SA (1 MBq/μg; ~ 144 MBq/nmole). The SA of panitumumab-DOTA-[^177^Lu]Lu was also identical to that used in our previous study of RIT with panitumumab-MCP-[^177^Lu]Lu (0.6 MBq/μg; ~ 87 MBq/nmole) (Aghevlian et al., 2019).

Panitumumab-DOTA-[^111^In]In appeared to provide greater tumour growth inhibition in NOD/SCID mice with s.c. PANC-1 tumours (Fig. 4a) than panitumumab-DOTA-[^177^Lu]Lu in NRG mice with PANC-1 tumours (Fig. 4b). However, since a total of 30 MBq (30 μg; ~ 0.21 nmoles) of panitumumab-DOTA-^111^In was administered vs. 6.0 MBq (10 μg; ~ 0.07 nmoles) of panitumumab-DOTA-^177^Lu (Scheme 1), panitumumab-DOTA-^111^In may be less effective per MBq administered than panitumumab-DOTA-[^177^Lu]Lu. Since NOD/SCID mice were used to engraft PANC-1 tumours for RIT with panitumumab-MCP-[^111^In]In or panitumumab-DOTA-[^111^In]In and NRG mice for panitumumab-DOTA-[^177^Lu]Lu, it is difficult to make direct comparisons of the effectiveness of ^111^In or ^177^Lu-labeled RICs. PANC-1 tumours grew more slowly in NOD/SCID mice than in NRG mice, revealed by the slower tumour growth rates in mice treated with normal saline or unlabeled panitumumab (Fig. 4a,b). Thus, it is possible that panitumumab-DOTA-[^177^Lu]Lu may be even more effective than panitumumab-MCP-[^111^In]In or panitumumab-DOTA-[^111^In]In if administered to NOD/SCID mice, but our concern was that panitumumab-DOTA-[^177^Lu]Lu may be unusually toxic in these mice since NOD/SCID mice harbour a germ-line defect in DNA repair that radiosensitizes normal tissues (Biedermann et al., 1991). Nonetheless, by increasing the amount administered by 5-fold, apparently greater tumour growth inhibition was achieved in NOD/SCID mice treated with panitumumab-DOTA-[^111^In]In than panitumumab-DOTA-[^177^Lu]Lu, while comparable tumour growth inhibition was found for panitumumab-MCP-[^111^In]In. The TDT for NOD/SCID mice with s.c. PANC-1 xenografts that received a total amount of 30.0 MBq (30 μg; ~ 0.21 nmoles) of panitumumab-DOTA-[^111^In]In or panitumumab-MCP-[^111^In]In were 51.8 days and 28.1 days, respectively, which were 2.5-fold and 1.3-fold longer, respectively, than in NRG mice treated with panitumumab-DOTA-[^177^Lu]Lu (20.9 days). In addition, the mean ± SD TGI in NOD/SCID mice at 43 days after administration of panitumumab-DOTA-[^111^In]In (4.0 ± 0.3) or panitumumab-MCP-[^111^In]In (3.0 ± 0.4), when at least 3 mice in each group remained alive, was similar to that at 33 days after treatment of NRG mice with a single amount of panitumumab-DOTA-[^177^Lu]Lu (2.9 ± 0.4).

We previously found that ^177^Lu-labeled bispecific radioimmunoconjugates (bsRICs) that bind HER2 and EGFR were more effective for RIT of s.c. MDA-MB-231/H2N human breast cancer xenografts in athymic mice than the corresponding ^111^In-labeled bsRICs when administered at the same amounts (11.0 MBq) (Razumienko et al., 2016). However, the long range β-particles emitted by ^177^Lu increase the risk for hematopoietic system toxicity mediated by a “cross-fire” effect and this has been dose-limiting for RIT with ^177^Lu-labeled RICs (Vallabhajosula et al., 2016). Administration of higher amounts of ^177^Lu-labeled panitumumab to increase the effectiveness for RIT of PnCa would result in increased normal tissue toxicity. Alternatively, higher amounts of ^111^In-labeled panitumumab could be administered to achieve greater therapeutic effects, due to the absence of a cross-fire effect in the case of AE (Ku et al., 2019). Interestingly, Behr et al. (Behr et al., 2000) reported that CO17-1A monoclonal antibodies labeled with the AE-emitters, ^125^I or ^111^In were more effective for RIT of human colon cancer xenografts in mice than CO171A labeled with the β-particle emitters, ^131^I or ^90^Y, but the RICs were administered at equitoxic and not equal amounts. Notably, a 10-fold higher amount of ^125^I-CO17-1A (111 MBq) than ^131^I-CO17-1A (11.1 MBq) and a 21-fold higher amount of ^111^In-CO-17-1A (85 MBq) than ^90^Y-CO17-1A (4 MBq) was administered safely for these RIT studies. Nonetheless, ^111^In emits γ-photons [Eγ-171 keV (90%) and 245 keV (94%)] that irradiate normal tissues, and at the high amounts needed for RIT, may cause significant non-targeted normal tissue toxicity. AE emitters with a higher ratio of electrons/photons such as ^201^Tl, ^193m^Pt, ^195m^Pt, ^197^Hg, ^119^Sb or ^161^Tb may reduce the potential for γ-photon mediated toxicity from RIT (Ku et al., 2019).

## Conclusions

Panitumumab labeled with AE-emitting, ^111^In was less potent in vitro on a per MBq exposure basis for killing PANC-1 cells in vitro than panitumumab-labeled with β-particle emitting, ^177^Lu. This lower cytotoxicity was correlated with fewer DNA DSBs in the cell nucleus and a lower absorbed dose in the nucleus. RIT with panitumumab labeled with ^111^In or ^177^Lu was effective for inhibiting the growth of s.c. PANC-1 xenografts in vivo in NOD/SCID or NRG mice, respectively, at amounts that caused no normal tissue toxicity, while unlabeled panitumumab was ineffective. However, a 5-fold higher amount of ^111^In-labeled panitumumab (total = 30 MBq; 30 μg; ~ 0.21 nmoles) than ^177^Lu-labeled panitumumab (6 MBq; 10 μg; ~ 0.07 nmoles) was required to achieve equivalent or greater tumour growth inhibition. Nonetheless, we conclude that RIT of PnCa with ^111^In or ^177^Lu-labeled panitumumab exploiting EGFR overexpression present in > 90% of cases is a promising approach that could overcome the resistance seen with treatment with anti-EGFR monoclonal antibodies such as panitumumab.

## Supplementary Information

**Additional file 1: Fig. S1.** (a) Percent cell bound radioactivity at selected times after incubation of 2 × 10^5^ PANC-1 cells with 1.2 MBq (2.5 nmoles/L) of panitumumab-DOTA-[^177^Lu]Lu, panitumumab-DOTA-[^177^Lu]Lu combined with an excess of unlabeled panitumumab, or non-specific hIgG-DOTA-[^177^Lu]Lu. (b) Percent of cell bound radioactivity at selected times on the cell membrane, internalized into the cytoplasm or transported to the nucleus in PANC-1 cells incubated with panitumumab-DOTA-^177^Lu. The time-integrated radioactivity (Bq × sec) in each subcellular compartment (Ã_s_) was calculated and used to estimate the absorbed doses in the nucleus as described in the Methods of the main article and shown in the Results (Table 1). **Fig. S2.** Radioactivity vs. time in the tumor and normal organs in NOD/SCID mice with s.c. PANC-1 xenografts injected i.v. (tail vein) with (a) panitumumab-DOTA-[^111^In]In or (b) panitumumab-MCP-[^111^In]In, or (c) in NRG mice with s.c. PANC-1 xenografts injected with panitumumab-DOTA-[^177^Lu]Lu. The time-integrated radioactivity (Bq × sec) in the tumor and source organs (Ã_s_) was obtained by integration and used to estimate the absorbed doses in the tumor and normal organs as described in the Methods of the main article and shown in the Results (Table 2).

## Data Availability

All data generated or analyzed during this study are included in this published article [and its supplementary information files].

## Abbreviations

AE: Auger electron
ALT: Alanine transaminase
ATCC: American type culture collection
AUC: Area under the curve
Bq: Becquerel
BWI: Body weight index
CBC: Complete blood cell count
CM: Cell membrane
Cy: Cytoplasm
Ci: Curie
Cr: Creatinine
Cs: Cell survival
CT: Computed tomography
Da: Dalton
DMEM: Dulbecco’s Modified Eagle’s Medium
DNA: Deoxyribonucleic acid
DOTA: 1 4 7 10-tetraazacyclododecane tetraacetic acid
DOTA-NHS-ester: 1,4,7,10-Tetraazacyclododecane-1,4,7,10-tetraacetic acid mono-N hydroxysuccinimide ester
DSB: DNA double strand breaks
EC: Electron capture
EGFR: Epidermal Growth Factor Receptor
eV: Electron volt
FBS: Fetal bovine serum
Gy: Gray
Hb: Hemoglobin
HPLC: High performance liquid chromatography
HyNic-MCP: Hydrazino-nicotinamide Metal Chelating Polymer
γ-H2AX: Gamma histone-2AX
i.v.: Intravenous
^111^In: Indium-111
ID/g: Injected dose per gram
IgG: Immunoglobulin G
ITLC: Instant thin layer chromatography
^177^Lu: Lutetium-177
LET: Linear Energy Transfer
MBq: Mega Becquerel
MCNP: Monte Carlo N-Particle
MWCO: Molecular weight cut off
N: Nucleus
NHS: N-Hydroxysuccinimide
NLS: Nuclear localizing sequence peptide
OLINDA/EXM: Organ Level INternal Dose Assessment/EXponential Modeling
p.i.: Post injection
PBS: Phosphate-buffered saline
PE: Plating efficiency
PEG: Polyethylene glycol
PnCa: Pancreatic Cancer
RBC: Red blood cell
RIC: Radioimmunoconjugate
RIT: Radioimmunotherapy
s.c: Subcutaneously
SD: Standard deviation
SDS-PAGE: Sodium Dodecyl Sulfate Polyacrylamide Gel Electrophoresis
SEC: Size exclusion column
SF: Surviving Fraction
SPECT: Single-photon emission computed tomography
TDT: Tumour doubling time
TGI: Tumour growth index
WBC: White blood cell

## References

[CR1] Aghevlian S, Cai Z, Lu Y, Hedley DW, Winnik MA, Reilly RM (2019). Radioimmunotherapy of PANC-1 human pancreatic cancer xenografts in NRG mice with panitumumab modified with metal-chelating polymers complexed to ^177^Lu. Mol Pharm.

[CR2] Aghevlian S, Lu Y, Winnik MA, Hedley DW, Reilly RM (2018). Panitumumab modified with metal-chelating polymers (MCP) complexed to ^111^In and ^177^Lu-an EGFR-targeted theranostic for pancreatic cancer. Mol Pharm.

[CR3] Anonymous (1995). Drugs directorate guidelines for Toxicologic evaluation.

[CR4] Baranyai Z, Tircsó G, Rösch F. The use of the macrocyclic chelator DOTA in radiochemical separations. Eur J Inorg Chem. 2020;1:36–56.

[CR5] Behr TM, Béhé M, Löhr M, Sgouros G, Angerstein C, Wehrmann E (2000). Therapeutic advantages of auger electron- over beta-emitting radiometals or radioiodine when conjugated to internalizing antibodies. Eur J Nucl Med.

[CR6] Biedermann KA, Sun JR, Giaccia AJ, Tosto LM, Brown JM (1991). Scid mutation in mice confers hypersensitivity to ionizing radiation and a deficiency in DNA double-strand break repair. Proc Natl Acad Sci U S A.

[CR7] Bitar A, Lisbona A, Thedrez P, Sai Maurel C, Le Forestier D, Barbet J (2007). A voxel-based mouse for internal dose calculations using Monte Carlo simulations (MCNP). Phys Med Biol.

[CR8] Cai Z, Chen Z, Bailey KE, Scollard DA, Reilly RM, Vallis KA (2008). Relationship between induction of phosphorylated H2AX and survival in breast cancer cells exposed to ^111^In-DTPA-hEGF. J Nucl Med.

[CR9] Cai Z, Kwon YL, Reilly RM (2017). Monte Carlo N-particle (MCNP) modeling of the cellular dosimetry of ^64^Cu: comparison with MIRDcell S values and implications for studies of its cytotoxic effects. J Nucl Med.

[CR10] Cai Z, Pignol JP, Chan C, Reilly RM (2010). Cellular dosimetry of ^111^In using monte carlo N-particle computer code: comparison with analytic methods and correlation with in vitro cytotoxicity. J Nucl Med.

[CR11] Cai Z, Vallis KA, Reilly RM (2009). Computational analysis of the number, area and density of gamma-H2AX foci in breast cancer cells exposed to ^111^In-DTPA-hEGF or gamma-rays using image-J software. Int J Radiat Biol.

[CR12] Cleary SP, Gryfe R, Guindi M, Greig P, Smith L (2004). Prognostic factors in resected pancreatic adenocarcinoma: analysis of actual 5-year survivors. J Am Coll Surg.

[CR13] Conroy T, Desseigne F, Ychou M, Bouche O, Guimbaud R (2011). FOLFIRINOX versus gemcitabine for metastatic pancreatic cancer. N Engl J Med.

[CR14] Costantini DL, Chan C, Cai Z, Vallis KA, Reilly RM (2007). ^111^In-labeled trastuzumab (Herceptin) modified with nuclear localization sequences (NLS): an auger electron-emitting radiotherapeutic agent for HER2/neu-amplified breast cancer. J Nucl Med.

[CR15] Crane CH, Varadhachary GR, Yordy JS, Staerkel GA, Javle MM (2011). Phase II trial of cetuximab, gemcitabine, and oxaliplatin followed by chemoradiation with cetuximab for locally advanced (T4) pancreatic adenocarcinoma: correlation of Smad4(Dpc4) immunostaining with pattern of disease progression. J Clin Oncol.

[CR16] Eckerman KF, Endo A (2008). MIRD: radionuclide data and decay schemes.

[CR17] Eser S, Schnieke A, Schneider G, Saur D (2014). Oncogenic KRAS signalling in pancreatic cancer. Br J Cancer.

[CR18] Euhus DM, Hudd C, LaRegina MC, Johnson FE (1986). Tumor measurement in the nude mouse. J Surg Oncol.

[CR19] Goddu SM, Howell RW, Rao DV (1994). Cellular dosimetry: absorbed fractions for monoenergetic electron and alpha particle sources and S-values for radionuclides uniformly distributed in different cell compartments. J Nucl Med.

[CR20] Halfdanarson TR, Foster NR, Kim GP, Meyers JP, Smyrk (2019). A phase II randomized trial of panitumumab, erlotinib, and gemcitabine versus erlotinib and gemcitabine in patients with untreated, metastatic pancreatic adenocarcinoma: north central cancer treatment group trial N064B (Alliance). Oncologist.

[CR21] Kamisawa T, Wood LD, Itoi T, Takaori K (2016). Pancreatic cancer. Lancet 2019.

[CR22] Korc M, Meltzer P, Trent J (1986). Enhanced expression of epidermal growth factor receptor correlates with alterations of chromosome 7 in human pancreatic cancer. Proc Natl Acad Sci U S A.

[CR23] Ku A, Facca VJ, Cai Z, Reilly RM (2019). Auger electrons for cancer therapy - a review. EJNMMI Radiopharm Chem.

[CR24] Liao HJ, Carpenter G (2009). Cetuximab/C225-induced intracellular trafficking of epidermal growth factor receptor. Cancer Res.

[CR25] Liu Z, Ma T, Liu H, Jin Z, Sun X (2014). ^177^Lu-labeled antibodies for EGFR-targeted SPECT/CT imaging and radioimmunotherapy in a preclinical head and neck carcinoma model. Mol Pharm.

[CR26] Lo HW, Hsu SC, Hung MC (2006). EGFR signaling pathway in breast cancers: from traditional signal transduction to direct nuclear translocation. Breast Cancer ResTreat.

[CR27] Lu Y, Boyle AJ, Cao P-J, Hedley D, Reilly RM, Winnik MA (2017). EGFR-targeted metal chelating polymers (MCPs) harboring multiple pendant PEG2K chains for microPET/CT imaging of patient-derived pancreatic cancer xenografts. ACS Biomater Sci Eng.

[CR28] Ma Y, Guo FC, Wang W, Shi HS, Li D, Wang YS (2013). Kras gene mutation as a predictor of cancer cell responsiveness to metformin. Mol Med Rep.

[CR29] Ochakovskaya R, Osorio L, Goldenberg DM, Mattes MJ (2001). Therapy of disseminated B-cell lymphoma xenografts in severe combined immunodeficient mice with an anti-CD74 antibody conjugated with ^111^indium, ^67^gallium, or ^90^yttrium. Clin Cancer Res.

[CR30] Paillas S, Ladjohounlou R, Lozza C, Pichard A, Boudousq V (2016). Localized irradiation of cell membrane by auger electrons is cytotoxic through oxidative stress-mediated nontargeted effects. Antioxid Redox Signal.

[CR31] Razumienko EJ, Chen JC, Cai Z, Chan C, Reilly RM (2016). Dual-receptor-targeted radioimmunotherapy of human breast cancer xenografts in athymic mice coexpressing HER2 and EGFR using ^177^Lu- or ^111^In-labeled bispecific radioimmunoconjugates. J Nucl Med.

[CR32] Reilly RM, Knaeblein J, Mueller R (2005). Biomolecules as targeting vehicles for in situ radiotherapy of malignancies. Modern biopharmaceuticals: design, development and optimization.

[CR33] Stabin MG, Sparks RB, Crowe E (2005). OLINDA/EXM: the second-generation personal computer software for internal dose assessment in nuclear medicine. J Nucl Med.

[CR34] Tabrizi MA, Tseng CM, Roskos LK (2006). Elimination mechanisms of therapeutic monoclonal antibodies. Drug Discov Today.

[CR35] Troiani T, Martinelli E, Capasso A, Morgillo F, Orditura M, De Vita F (2012). Targeting EGFR in pancreatic cancer treatment. Curr Drug Targets.

[CR36] Vallabhajosula S, Nikolopoulou A, Jhanwar YS, Kaur G, Tagawa ST (2016). Radioimmunotherapy of metastatic prostate cancer with ^177^Lu-DOTAhuJ591 anti prostate specific membrane antigen specific monoclonal antibody. Curr Radiopharm.

[CR37] Weber T, Botticher B, Mier W, Sauter M, Kramer S (2016). High treatment efficacy by dual targeting of Burkitt's lymphoma xenografted mice with a ^177^Lu-based CD22-specific radioimmunoconjugate and rituximab. Eur J Nucl Med Mol Imaging.

