# Supplementary material for: Radioimmunotherapy of PANC-1 human pancreatic cancer xenografts in NOD/SCID or NRG mice with Panitumumab labeled with Auger electron emitting, 111In or β-particle emitting, 177Lu
Source: EJNMMI Radiopharm Chem. 2020 Nov 9;5:22. doi: 10.1186/s41181-020-00111-y (PMC7652961; doi:10.1186/s41181-020-00111-y)
Supplement: Supplementary file 1 — Additional file 1: Fig. S1. (a) Percent cell bound radioactivity at selected times after incubation of 2 × 10^5^ PANC-1 cells with 1.2 MBq (2.5 nmoles/L) of panitumumab-DOTA-[177Lu]Lu, panitumumab-DOTA-[177Lu]Lu combined with an excess of unlabeled panitumumab, or non-specific hIgG-DOTA-[177Lu]Lu. (b) Percent of cell bound radioactivity at selected times on the cell membrane, internalized into the cytoplasm or transported to the nucleus in PANC-1 cells incubated with panitumumab-DOTA-177Lu. The time-integrated radioactivity (Bq × sec) in each subcellular compartment (Ãs) was calculated and used to estimate the absorbed doses in the nucleus as described in the Methods of the main article and shown in the Results (Table 1). Fig. S2. Radioactivity vs. time in the tumor and normal organs in NOD/SCID mice with s.c. PANC-1 xenografts injected i.v. (tail vein) with (a) panitumumab-DOTA-[111In]In or (b) panitumumab-MCP-[111In]In, or (c) in NRG mice with s.c. PANC-1 xenografts injected with panitumumab-DOTA-[177Lu]Lu. The time-integrated radioactivity (Bq × sec) in the tumor and source organs (Ãs) was obtained by integration and used to estimate the absorbed doses in the tumor and normal organs as described in the Methods of the main article and shown in the Results (Table 2). [file 41181_2020_111_MOESM1_ESM.docx]

**Radioimmunotherapy of PANC-1 Human Pancreatic Cancer Xenografts in NOD/SCID or NRG Mice with Panitumumab Labeled with Auger Electron Emitting, ^111^In or β-Particle Emitting, ^177^Lu**

Sadaf Aghevlian^1^, Zhongli Cai^1^, David Hedley^,3^, Mitchell A. Winnik^2^

and Raymond M. Reilly^1,4,*^

^1^ Department of Pharmaceutical Sciences, University of Toronto, Toronto, ON, Canada

^2^ Department of Chemistry, University of Toronto, Toronto, ON, Canada

^3^ Department of Medical Oncology, Princess Margaret Cancer Centre, University Health Network, Toronto, ON, Canada

^4^ Department of Medical Imaging, University of Toronto, Toronto, ON, Canada

**Supplementary Information**


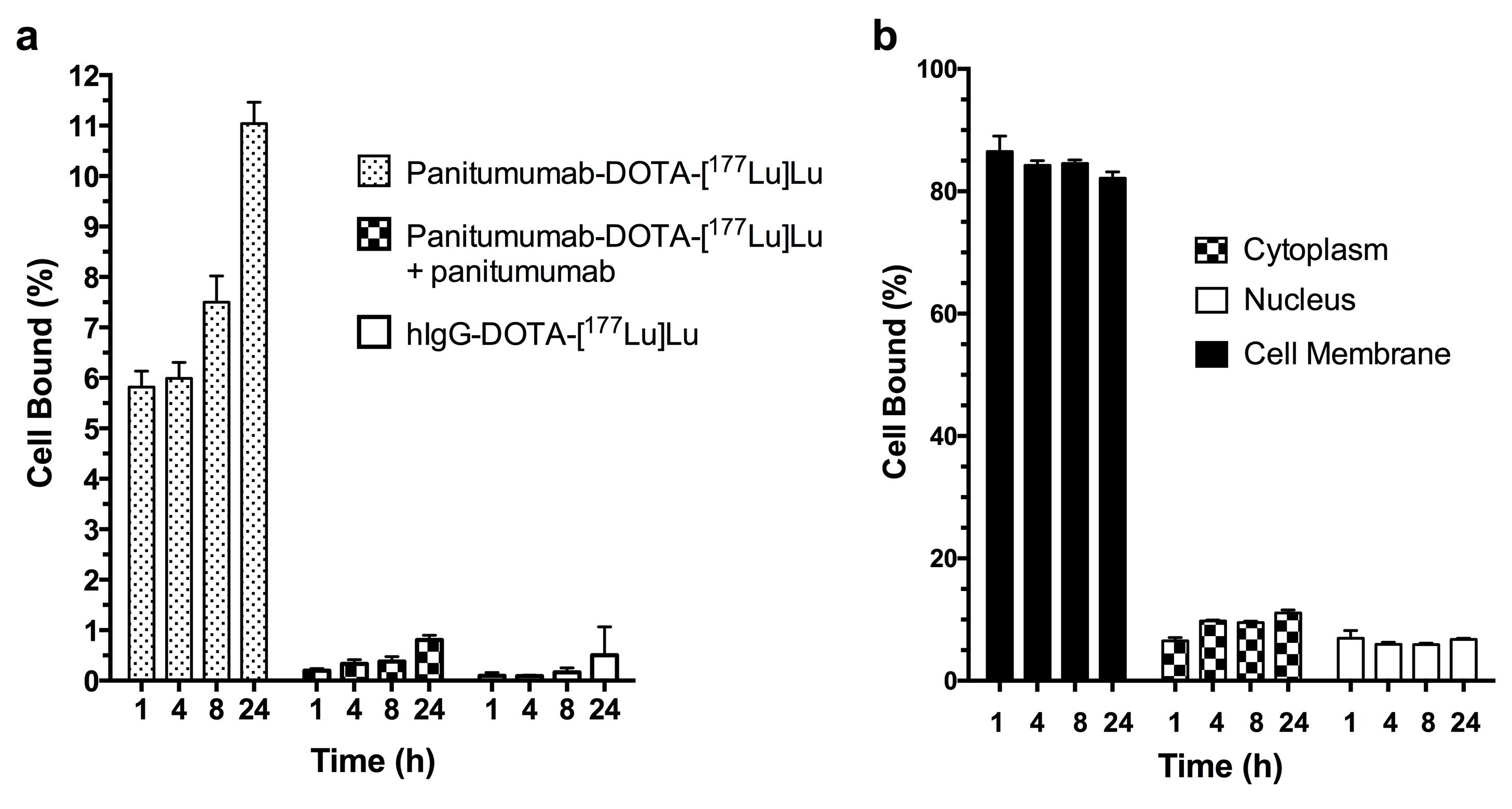


**Fig. S1.** (a) Percent cell bound radioactivity at selected times after incubation of 2 × 10^5^ PANC-1 cells with 1.2 MBq (2.5 nmoles/L) of panitumumab-DOTA-[^177^Lu]Lu, panitumumab-DOTA-[^177^Lu]Lu combined with an excess of unlabeled panitumumab, or non-specific hIgG-DOTA-[^177^Lu]Lu. (b) Percent of cell bound radioactivity at selected times on the cell membrane, internalized into the cytoplasm or transported to the nucleus in PANC-1 cells incubated with panitumumab-DOTA-^177^Lu. The time-integrated radioactivity (Bq × sec) in each subcellular compartment ($\tilde{A}_{S}$) was calculated and used to estimate the absorbed doses in the nucleus as described in the Methods of the main article and shown in the Results (**Table 1**).


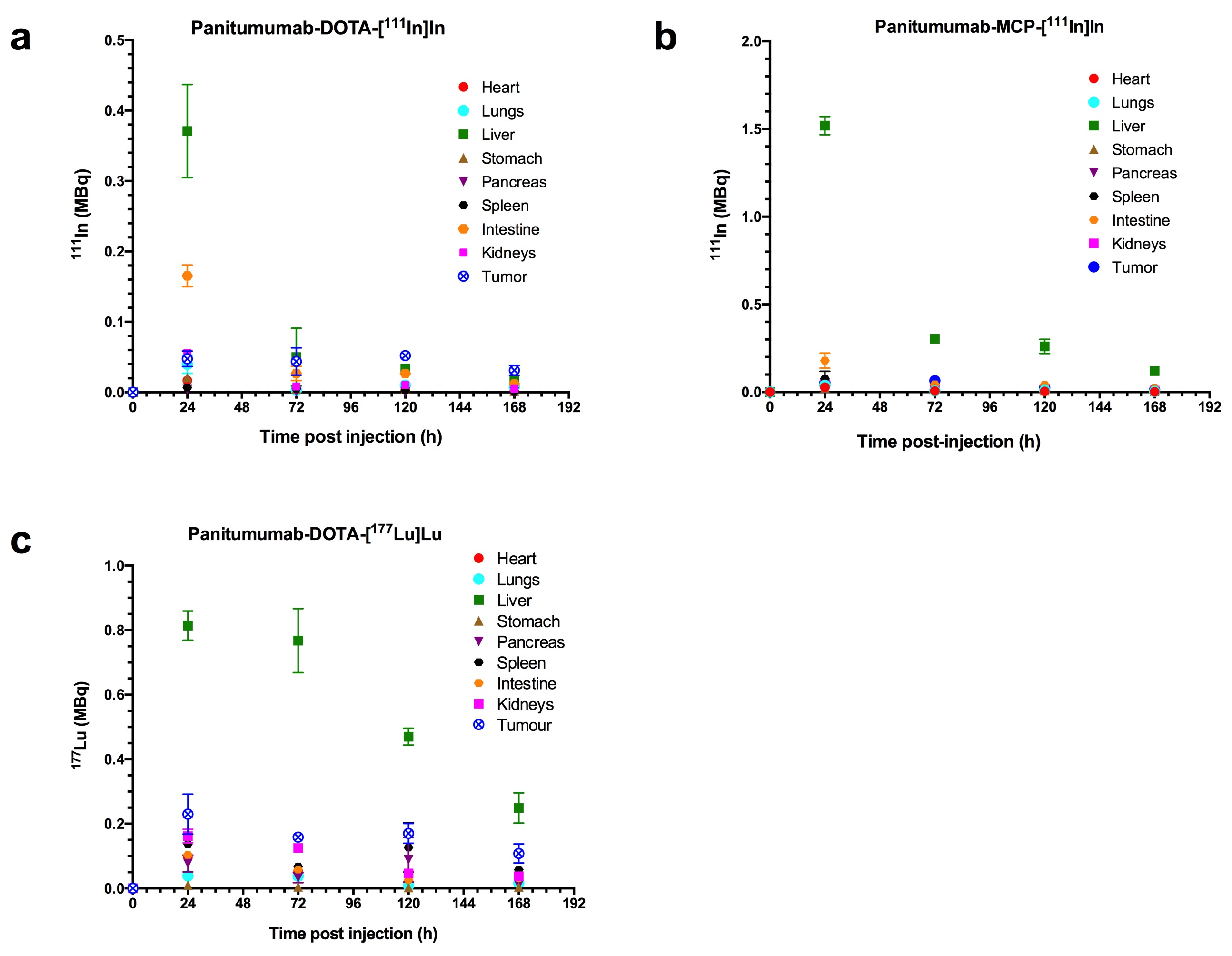


**Fig. S2.** Radioactivity vs. time in the tumor and normal organs in NOD/SCID mice with s.c. PANC-1 xenografts injected i.v. (tail vein) with (a) panitumumab-DOTA-[^111^In]In or (b) panitumumab-MCP-[^111^In]In, or (c) in NRG mice with s.c. PANC-1 xenografts injected with panitumumab-DOTA-[^177^Lu]Lu. The time-integrated radioactivity (Bq × sec) in the tumor and source organs ($\tilde{A}_{S}$) was obtained by integration and used to estimate the absorbed doses in the tumor and normal organs as described in the Methods of the main article and shown in the Results (Table 2).
